# Age threshold for recommending higher protein intake to prevent age-related muscle weakness: A cross-sectional study in Japan

**DOI:** 10.1371/journal.pone.0208169

**Published:** 2018-12-12

**Authors:** Hitomi Suga, Hideki Hashimoto

**Affiliations:** 1 Department of Social and Preventive Epidemiology, School of Public Health, The University of Tokyo, Bunkyo-ku, Tokyo, Japan; 2 Departments of Health and Social Behavior, School of Public Health, The University of Tokyo, Bunkyo-ku, Tokyo, Japan; Ehime University Graduate School of Medicine, JAPAN

## Abstract

Although insufficient dietary protein intake is a known risk factor for age-related muscle weakness, the optimal age at which higher protein intake is required to prevent muscle weakness is yet to be determined. Using a population-based panel survey of community-dwelling people aged 50–75 years, this cross-sectional study aimed to find the age threshold at which a higher protein intake is associated with higher muscle strength. We utilized a dataset from the Japanese Study of Aging and Retirement conducted between 2007 and 2011. Dietary protein intake was estimated using a validated dietary questionnaire and energy-adjusted via density method. Grip strength was measured using a Smedley-type handheld dynamometer. We calculated the marginal effect (and 95% confidence intervals) of protein intake on grip strength with stratification by age using multiple linear regression analyses with robust variance adjusting for potential confounders. There were 9,485 observations from 5,790 participants in the final analysis. Marginal effects of protein intake on grip strength increased with age, and it reached significance and had a positive impact only among men aged ≥75 years and women aged ≥65 years. With an additional 1% energy of protein intake, grip strength was increased by 0.10 kg and 0.19 kg for men and women aged ≥75 years, respectively. Our result indicated the possibility that women needed a high protein intake from a younger age compared with men. Further studies are needed to clarify from when a higher protein intake is recommended to prevent muscle weakness.

## Introduction

Age-related loss of muscle strength is a characteristic feature of sarcopenia [1], which is in turn related to falls [2,3], low physical performance [3], prolonged hospital stay [4], and increased healthcare and hospitalization costs [5,6]. Age-related loss of muscle strength starts around the fourth decade of life [7,8], and its early prevention is an important target of public health intervention in the ageing population.

Insufficient dietary protein intake is a cause of muscle weakness in elderly individuals [9]. Protein is a component of the skeletal muscle, and its intake stimulates muscle protein synthesis in humans [10]. Experimental studies reported that the response of muscle protein anabolism to dietary protein is impaired with age and that elderly individuals require more dietary protein to maintain nitrogen balance and to stimulate muscle protein synthesis than the young [10–14]. The protein intake requirement is further increased due to both acute and chronic diseases that are more prevalent among older adults [15–17]. Therefore, health authorities in some countries have adopted higher protein requirement values for elderly individuals in the Dietary Reference Intakes (DRI), including in Japan [18–22].

However, the age by which the protein intake requirement increases to maintain muscle protein remains unclear. Currently, the age threshold by which a higher protein intake is recommended in DRI varies across countries; it starts from age ≥75 years in France [20], from age ≥70 years in Australia, New Zealand, and Japan [19,21]; and from age ≥65 years in Germany, Austria, Switzerland, Denmark, Finland, Iceland, Norway, and Sweden [18,22]. In the present study, we aimed to find the age threshold at which a higher protein intake is associated with higher muscle strength using a population-based panel survey of community-dwelling people aged ≥50 years.

## Materials and methods

### Study population

We utilized a public-use dataset from the Japanese Study of Aging and Retirement (JSTAR) collected between 2007 and 2011. This dataset was a resampled dataset from the whole JSTAR dataset to protect confidential information. The JSTAR is a panel survey of community-dwelling middle-aged and elderly people aged 50–75 years at baseline and collects information on the economic, social, and health condition. The JSTAR was conducted by The Research Institute of Economy, Trade and Industry, Hitotsubashi University, and The University of Tokyo. The details of the sampling strategy were described previously [23]. The individuals aged 50–75 years were randomly sampled based on household registration in 10 municipalities across Japan [23]. The first-wave survey for five municipalities (Takikawa City, Sendai City, Adachi Ward, Kanazawa City, and Shirakawa Town) was conducted in 2007, for two municipalities (Naha City and Tosu City) in 2009, and for three municipalities (Chohu City, Hiroshima City, and Tondabayashi City) in 2011. Of the 15,752 eligible men and women identified in the ten municipalities, 7,914 men and women participated in the first-wave surveys [23–25]. The second- and third-wave surveys were conducted two years after the previous surveys. The current study was conducted according to the guidelines laid down in the Declaration of Helsinki, and the protocol of the JSTAR was approved by the internal review board of The University of Tokyo Graduate School of Medicine (approval no. 2676). Written consent was obtained from each participant at the time of recruitment of JSTAR survey.

### Dietary assessments

Dietary protein and energy intakes during the previous month were assessed using a brief-type self-administered diet history questionnaire (BDHQ) in each survey. Details of the BDHQ were described in a previous report [26]. Briefly, the BDHQ is a four-page fixed-portion questionnaire that assesses the consumption frequency of 58 listed food items and beverages during the preceding month. The fixed portion size of the 58 food and beverage items was determined based on several Japanese recipe books [27]. Energy and protein intakes were estimated based on the frequency of consumption of each food and beverage, presumed portion size, and energy and protein contents derived from the Standard Tables of Food Composition in Japan by using *ad hoc* computer algorithm for the BDHQ [28]. The validity of nutrient intake estimated via the BDHQ was assessed using the average nutrient intake calculated from a 16-day dietary record among 184 Japanese adults (92 men aged 32–76 years and 92 women aged 31–69 years) as a reference [26]. The Pearson correlation coefficients for energy and energy-adjusted protein intake between the BDHQ and the average of the 16-day dietary record were 0.23 and 0.38 for men and 0.29 and 0.35 for women, respectively [26]. Protein intake values that were energy-adjusted using a density method (% energy) was used for the analysis to minimize the influence of dietary misreporting [29].

### Grip strength

We used grip strength as an indicator of the participants’ muscle strength for the analysis. The grip strength of the dominant hand in kilograms was measured with a portable handheld dynamometer (Smedley-type Hand Grip Meter, No. 6103; TANITA, Tokyo, Japan) in each wave of the survey. The participants stood with their arms parallel to the side of the body and squeezed the dynamometer as hard as possible in accordance with the original measurement style for a Smedley-type dynamometer [23]. In this survey, grip strength was measured once to reduce the effect of muscle fatigue on the measurement of grip strength. A second measurement was conducted only when the first one failed.

### Other variables

Age, sex, body weight, and height were self-reported as parts of the BDHQ. Body mass index (BMI) was calculated as body weight (kg) divided by body height (m) squared. Self-perceived health status was reported using six categories: excellent, very good, good, fair, poor, and do not know. Limitation in instrumental activities of daily living (IADL) was defined as having difficulties in one or more of the 15 types of daily activities and was measured using the Tokyo Metropolitan Institute of Gerontology Index of Competence [30], asking whether participants have difficulties in 15 types of daily activities (e.g., using public transportation, shopping for daily necessities, preparing meals, paying bills, and visiting homes of friends). Limitation in mobility was determined based on difficulty in at least one or more items of mobility (e.g., sitting in a chair for 2 hours, walking for 100 m, and going upstairs without using a handrail).

### Statistical analysis

All statistical analyses were conducted separately for men and women using STATA statistical software package version 14 (STATA Corp., College Station, TX). The baseline characteristics of the participants were compared among those in their 50s, 60s, and 70s using analysis of variance for continuous variables and the chi-square test for categorical variables. Also, we calculated the percentage of participants whose protein intake was lower than the lowest limit of recommended protein intake (13% energy) according to the 2015 DRI for Japanese [19]. To examine the effect of protein intake on muscle strength, we calculated the marginal effect of protein intake on grip strength (that is, how much grip strength was changed by an additional 1% energy of protein intake) at the average of the covariates in each age strata using multiple linear regression analyses with robust variance. As covariates for adjustment, we used variables for limitation in IADL (yes or no), limitation in mobility (yes or no), self-perceived health status (six categories), BMI (kg/m^2^, continuous), survey year (three categories), and total energy intake (kcal/day, continuous). To determine the age at which protein intake was positively associated with grip strength, we stratified the participants aged 53–74 years by two-year age groups. Participants aged 50–52 years and ≥75 years were re-categorized into each age group (aged ≤52 years and ≥75 years) because the sample size of the participants aged 50–52 years and ≥75 years was relatively small.

We included all the observations obtained from the three-wave panel surveys. Of the 12,143 observations derived from 6,815 participants, we excluded observations with missing data for age (n = 28), body height and weight (n = 5), grip strength (n = 1,106), self-reported health status (n = 593), and answers for IADL or mobility (n = 40). We also excluded those aged <50 years (n = 2), those with extremely high (>4000 kcal/day) or low (<500 kcal/day) (n = 99) energy intakes, and those with history of cerebrovascular diseases, arthritis, or Parkinson’s disease (n = 785) because these diseases and their sequelae can weaken grip strength regardless of the amount of dietary protein intake. Ultimately, 9,485 observations were derived from the 5,790 participants that remained for the final analysis.

## Results

The mean (± SD) age of the participants in the first-wave survey was 62.4 ± 7.0 years, and 46.6% were men. Among the 9,485 observations, 6,334 (66.8%), 1,703 (18.0%), and 1,448 (15.3%) were obtained from municipalities where the first-wave survey was conducted in 2007, 2009, and 2011, respectively. A total of 2,037 (21.5%) observations were classified as protein intake lower than the recommended limit (13% energy) [19]. The baseline characteristics of the participants among the 10-year age groups are shown in **Table 1**. The participants in their 70s were less likely to have protein intake lower than the recommended limit (*p*<0.0001) [19], but they were also more likely to have limitations in IADL and mobility (*p*<0.0001), reported poorer self-perceived health status (*p*<0.0001), and had lower grip strength (*p*<0.0001). Energy and carbohydrate intake for men aged ≥70 years was significantly lower than that for men aged 50–59 years. Total fat intake for women aged ≥70 years was significantly lower than that for women aged 50–59 years.

**Table 1:** Baseline characteristics of the study population.

	50–59 years old		60–69 years old		70 years old and above		*p* ^a^
	Mean, SD or n (%)		Mean, SD or n (%)		Mean, SD or n (%)		
**Men (n = 4,571)**	(n = 1,496)		(n = 1,904)		(n = 1,171)		
Low protein intake, n (%) ^b^	587	(39.2)	537	(28.2)	250	(21.4)	< .0001
Body mass index, kg/m^2^	23.5	3.0	23.5	2.9	23.4	3.0	0.20
Year of survey, n (%)							< .0001
2007	580	(38.8)	562	(29.5)	267	(22.8)	
2009	490	(32.8)	545	(28.6)	320	(27.3)	
2011	426	(28.5)	797	(41.9)	584	(49.9)	
Residence							0.001
Five municipalities ^c^	1051	(70.3)	1311	(68.9)	826	(70.5)	
Two municipalities ^d^	257	(17.2)	295	(15.5)	219	(18.7)	
Three municipalities ^e^	188	(12.6)	298	(15.7)	126	(10.8)	
Self-perceived health status, n (%)							< .0001
Excellent	100	(6.7)	146	(7.7)	56	(4.8)	
Very good	384	(25.7)	543	(28.5)	283	(24.2)	
Good	866	(57.9)	1,051	(55.2)	690	(58.9)	
Fair	101	(6.8)	125	(6.6)	121	(10.3)	
Poor	5	(0.3)	9	(0.5)	(5.0)	(0.4)	
Do not know	40	(2.7)	30	(1.6)	(16.0)	(1.4)	
Limitation in instrumental activities of daily living, n (%)							< .0001
Yes	639	(42.7)	883	(46.4)	608	(51.9)	
Limitation in mobility, n (%)							< .0001
Yes	76	(5.1)	180	(9.5)	253	(21.6)	
Grip strength, kg	38.7	6.4	35.2	6.3	31.0	6.0	< .0001
Energy intake, kcal	2084	608	2036	611	2023	612	0.02
Protein intake, % energy	13.9	2.7	14.8	3.0	15.4	3.1	< .0001
Carbohydrate intake, % energy	62.5	7.5	61.1	8.1	60.5	8.1	< .0001
Total fat intake, % energy	23.7	5.5	24.1	5.8	24.1	5.9	0.06
**Women (n = 4,914)**	(n = 1,615)		(n = 2,055)		(n = 1,244)		
Low protein intake, n (%) ^b^	285	(17.7)	263	(12.8)	115	(9.2)	< .0001
Body mass index, kg/m^2^	22.6	3.3	23.0	3.5	23.1	3.5	< .0001
Year of survey, n (%)							< .0001
2007	548	(33.9)	587	(28.6)	301	(24.2)	
2009	505	(31.3)	561	(27.3)	320	(25.7)	
2011	562	(34.8)	907	(44.1)	623	(50.1)	
Residence							< .0001
Five municipalities ^c^	988	(61.2)	1300	(63.3)	858	(69.0)	
Two municipalities ^d^	323	(20.0)	376	(18.3)	233	(18.7)	
Three municipalities ^e^	304	(18.8)	379	(18.4)	153	(12.3)	
Self-perceived health status, n (%)							< .0001
Excellent	89	(5.5)	128	(6.2)	65	(5.2)	
Very good	408	(25.3)	544	(26.5)	274	(22.0)	
Good	976	(60.4)	1,235	(60.1)	760	(61.1)	
Fair	109	(6.8)	122	(5.9)	134	(10.8)	
Poor	2	(0.1)	4	(0.2)	5	(0.4)	
Do not know	31	(1.9)	22	(1.1)	6	(0.5)	
Limitation in instrumental activities of daily living, n (%)							
Yes	499	(30.9)	679	(33.0)	511	(41.1)	< .0001
Limitation in mobility, n (%)							
Yes	129	(8.0)	310	(15.1)	381	(30.6)	< .0001
Grip strength, kg	24.4	4.3	22.6	4.2	20.5	4.1	< .0001
Energy intake, kcal	1721	519	1735	530	1728	550	0.74
Protein intake, % energy	15.4	2.7	16.3	3.1	16.9	3.3	< .0001
Carbohydrate intake, % energy	56.4	7.3	56.5	7.6	56.7	7.9	0.62
Total fat intake, % energy	28.2	5.5	27.2	5.5	26.4	5.7	< .0001

SD: Standard deviations

^a^ p values were derived from ANOVA for continuous variables and chi-square test for categorical variables

^b^ The ratio of participants whose protein intake was lower than 13% energy.

^c^ Takikawa City, Sendai City, Adachi Ward, Kanazawa City, and Shirakawa Town

^d^ Naha City and Tosu City

^e^ Chohu City, Hiroshima City, and Tondabayashi City

The marginal effects of protein intake on grip strength at the average of the covariates in two-year age strata are shown in **Table 2**. After adjustment for all covariates, a higher protein intake associated with higher grip strength was noted for women aged ≥65 years. For men, this association was shown in the older age strata, that is, only those aged ≥75 years. The magnitude of these effects increased as age increased in women. Although positive associations were also shown in men aged 69–74 years and women aged 61–64 years, the associations were not statistically significant.

**Table 2:** Adjusted marginal effect for grip strength per unit of energy-adjusted protein intake.

	Men (n = 4,571)			Women (n = 4,914)		
Age	n	Regression coefficient (95% CI)		n	Regression coefficient (95% CI)	
50–52	260	-0.20	(-0.30, -0.10)	327	-0.11	(-0.20, -0.03)
53–54	271	-0.18	(-0.27, -0.09)	307	-0.09	(-0.17, -0.02)
55–56	337	-0.15	(-0.24, -0.07)	358	-0.07	(-0.13, 0.001)
57–58	387	-0.13	(-0.21, -0.05)	388	-0.04	(-0.10, 0.02)
59–60	454	-0.10	(-0.18, -0.03)	427	-0.02	(-0.07, 0.04)
61–62	369	-0.08	(-0.15, -0.01)	389	0.01	(-0.04, 0.05)
63–64	405	-0.06	(-0.12, 0.01)	479	0.03	(-0.02, 0.07)
65–66	365	-0.03	(-0.10, 0.03)	386	0.05	(0.01, 0.10)
67–68	350	-0.01	(-0.07, 0.06)	385	0.08	(0.03, 0.12)
69–70	417	0.02	(-0.05, 0.08)	429	0.10	(0.05, 0.15)
71–72	352	0.04	(-0.03, 0.11)	399	0.13	(0.07, 0.18)
73–74	332	0.06	(-0.01, 0.14)	329	0.15	(0.09, 0.21)
75≤	272	0.10	(0.02, 0.19)	311	0.19	(0.12, 0.26)

Regression coefficients represent the change in grip strength per 1% energy increase of protein intake.

Protein intake was energy-adjusted by using the density method (% energy).

Multiple linear regression model was used with adjustment for age, limitation in instrumental activities of daily living, limitation in mobility, self-perceived health status, BMI, survey year, and total energy intake.

## Discussion

We analyzed the data stratifying the participants into two-year age strata and found that higher protein intake became positively and significantly associated with higher grip strength for men aged ≥75 years and women aged ≥65 years.

Previous experimental studies have shown that the required protein intake for elderly individuals to meet nitrogen balance and to stimulate muscle protein synthesis is higher than that for the young [10–14]. Based on the results of experimental studies, elderly individuals have been encouraged to consume higher protein than the young [11,15,16]. Our results showed the positive effects of protein intake on grip strength only in older participants and supported the findings of the previous experimental studies.

Meanwhile, the results of epidemiological studies examining the association between protein intake and muscle strength are inconsistent. A cohort study conducted in the UK reported a positive association between protein intake and grip strength at baseline and at 1.5 years follow-up [31], and other cohort studies in the US also reported a positive association between protein intake and muscle strength at six years follow-up [32,33]. A cross-sectional study in Italy reported that participants with higher protein intake showed higher grip strength regardless of physical activity level [34]. By contrast, cohort studies conducted in Australia and Italy reported no significant association between protein intake and knee extension strength [35,36], and two cross-sectional studies in the US showed that protein intake had no significant effect on grip strength [37,38]. These inconsistent results among the previous studies may be due to differences in study design, study population, dietary assessment method, or indicator of muscle strength used in the studies. In addition, we speculated that these inconsistencies might be because the participants were not stratified by age.

A few epidemiological studies analyzed data stratifying participants by age. Mulla et al. reported no significant association between protein intake at age 36 and 43 years and grip strength at age 53 years [39]. Thompson et al. reported that protein intake had smaller effect on muscle strength of the leg flexors in middle-aged adults than in older adults [40]. Nevertheless, evidence that indicates the age threshold at which a higher protein intake is required to prevent the age-related loss of muscle strength is scarce. We found that higher protein intake is positively associated with higher grip strength for men aged ≥75 years and women aged ≥65 years. To the best of our knowledge, this study is the first to attempt to find evidence for the age threshold at which higher protein intake is recommended for the prevention of muscle weakness.

Meanwhile, our result showed that dietary protein intake had significant negative association with grip strength for men aged 50–62 years and women aged 50–24 years. Theoretically, it is unlikely that a higher protein intake has a negative effect on the maintenance of muscle strength for the middle-aged and the younger elderly. We speculate that a reverse causation may exist, or those with grip strength weaker for young age would take more protein-rich nutrition. This result implies that dietary intervention alone might be a less effective strategy to prevent age-related loss of muscle strength for middle-aged people. For the younger population, strategies other than dietary interventions to maintain muscle strength might be appropriate, such as exercise.

Another main finding was that the age at which the positive effect of a higher protein intake started to appear was higher in men than in women (≥75 years for men and ≥65 years for women). This result indicated the possibility that women needed a high protein intake from a younger age compared with men. Some previous studies also showed a sex difference in the association between protein intake and muscle strength, which was consistent with our results. The Hertfordshire Cohort Study reported that protein intake was inversely associated to grip strength in women but not in men [41]. Another cohort study showed that lower protein intake was associated with lower baseline grip strength only in women [31]. Since this study is cross-sectional, further studies are needed to clarify from when a higher protein intake is recommended to prevent muscle weakness.

We have two possible explanations for this sex difference. First, the response to ingested protein may be different by sex. An experimental study reported that the response of muscle protein anabolism that was stimulated by protein ingestion was lower in elderly women than in elderly men [42]. This implies that the negative effect of low protein intake is more significant for women than for men. Second, other factors, such as sex hormones, might have a stronger impact on the prevention of muscle weakness in men. Sex hormones have a beneficial effect on maintaining the muscle function [43]. Because testosterone decreases gradually from middle age while estrogen decreases sharply at menopause, age-related decrement in muscle strength might occur later in men than in women [43,44].

Our result showed that protein intake positively associated with grips strength for men aged ≥75 years and for women aged ≥65 years. However, it is difficult to determine whether very high protein intake (such as >30% energy) is recommended for higher muscle strength for the following reasons: First, the tolerable upper intake level of protein is not determined because the available evidence on the tolerable upper intake level of protein is not sufficient. Next, protein intake of many of our study population was less than 20% energy, and we do not have sufficient sample size to examine the effect of higher protein intake (such as >30% energy) on muscle strength in each age stratum. Further studies are needed to examine whether very high protein intake (such as >30% energy) is recommended for higher muscle strength using datasets that include sufficient number of participants with high protein intake.

The strength of this study is the use of a large sample of community-dwelling adults in a panel study that allows data analysis with stratification by 2-year age intervals. However, there are also limitations to our study. First, the cross-sectional study design does not allow assessment of causality because the temporality of the association is uncertain. Although we assessed the dietary habits of the participants for one month before grip strength was measured, the possibility of reverse causality cannot be excluded.

Second, the protein intake obtained from the BDHQ is an estimated protein intake. To minimize the effect of misreporting, we excluded participants whose energy intake was extremely low or high and used energy-adjusted values in the analysis [29]. Also, the validity of the BDHQ for estimating protein intake was limited. The validity of protein intake estimated from dietary questionnaires for Japanese tends to be lower than those for Westerners because of the complexity of the Japanese diet [45]. The median validity for estimated protein intake with 19 dietary questionnaires for Japanese was 0.39 [45]. Although the BDHQ seems to have an acceptable ability to estimate Japanese protein intake, the results should be interpreted with caution.

Third, several variables, such as body height, weight, and limitation of IADL or mobility, were based on self-reports, and thus misclassification may occur. In addition, because grip strength was measured only once, measurement error may occur.

Fourth, the response rate for the first-wave survey varied across cities (45.9%–87.8%) [23–25]. We could not include the city variable as a potential confounder in our analysis because the JSTAR’s public dataset did not include information on the cities where the participants lived. To reduce the effect of the difference in the response rate, we included the survey year variable into the multivariate analysis; however, this difference may bias the results.

Finally, there were some unavailable variables that might affect the association between protein intake and muscle strength. For example, we could not obtain the data on muscle mass, the presence of chronic kidney disease, and physical activity of participants. Although we adjusted for various potential confounding variables, the effects of unobserved confounders may not have been excluded because of these unobserved variables that can influence the maintenance of muscle strength and protein intake. To minimize the effect of unmeasured physical activity, we adjusted the protein intake value for energy intake, which is strongly correlated with physical activity [29]. Nevertheless, this may have been insufficient. Our result showed that the percentage of participants with low protein intake was lower among those in their 70s than those in their 50s. By contrast, older participants showed lower grip strength than younger participants. This implies that multiple factors (including protein intake) are associated with the maintenance of muscle strength. Further studies examining the association between protein intake and muscle strength including physical activity, muscle mass, and chronic kidney disease as covariates are also needed.

In conclusion, protein intake was significantly and positively associated with grip strength for men aged ≥75 years and for women aged ≥65 years, but not in middle-aged and early young-old adults. Further studies to determine the optimal age threshold for a nutritional strategy to prevent age-related muscle weakness are needed.
